# Identification of CD8^+^ T cell-related biomarkers and immune infiltration characteristic of rheumatoid arthritis

**DOI:** 10.18632/aging.205435

**Published:** 2024-01-16

**Authors:** Qizun Wang, Qianqian Li, Ronghuan Wang, Yanning Li, Jie Wang, Zhu Guo, Feng Li, Bohua Chen, Hongfei Xiang, Tianrui Wang, Xiaolin Wu

**Affiliations:** 1 Department of Orthopedics, The Affiliated Hospital of Qingdao University, Qingdao 266000, China; 2 Department of Ultrasound, The Affiliated Hospital of Qingdao University, Qingdao 266000, China; 3 Laboratory, The First Affiliated Hospital of Shanghai Jiao Tong University, Shanghai 200080, China; 4 Department of Orthopedics, Weifang People’s Hospital, Weifang, Shandong 261000, China; 5 Cancer Institute, The Affiliated Hospital of Qingdao University, Qingdao University, Qingdao Cancer Institute, Qingdao 266071, China

**Keywords:** rheumatoid arthritis, immune infiltration, CD8^+^ T cells, machine learning, personalized therapy

## Abstract

Rheumatoid arthritis (RA) is an autoimmune rheumatic disease, which do not respond well to current treatment partially. Therefore, further in-depth elucidation of the molecular mechanism and pathogenesis of RA is urgently needed for the diagnosis, personalized therapy and drug development. Herein, we collected 111 RA samples from Gene Expression Omnibus (GEO) database, and conducted differentially expressed genes and GESA analysis. Abnormal activation and imbalance of immune cells in RA were observed. WGCNA was utilized to explore the gene modules and CD8^+^ T cell-related genes (CRGs) were chosen for KEGG and GO analysis. Besides, to explore biomarkers of RA in depth, machine learning algorithms and bioinformatics analysis were used, and we identified GDF15, IGLC1, and IGHM as diagnostic markers of RA, which was confirmed by clinical samples. Next, ssGSEA algorithms were adopted to investigate the differences in immune infiltration of 23 immune cell subsets between RA and healthy control group. Finally, optimal classification analysis based on consensus clustering combined with ssGSEA algorithms were conducted. GDF15 was revealed that to be positively correlated with mast cells and type 2 T helper cells, but negatively correlated with most other immune cells. On the other hand, IGHM and IGLC1 were negatively correlated with CD56dim natural killer cells, while positively associated with other immune cells. Finally, RA samples in subtype A exhibited a higher immune infiltration status. This study could provide guidance for individualized treatment of RA patients and provide new targets for drug design.

## INTRODUCTION

Around 0.5% to 1% of the population worldwide is affected by rheumatoid arthritis (RA) [1] especially with pain in the hands and feet. RA is a chronic, systemic autoimmune disease associated with synovial tissue hyperplasia, synovial formation, cartilage destruction, and systemic complications [2]. RA causes joint inflammation and in severe cases can lead to permanent joint damage and disability. In addition, RA may affect other organs, including the lungs, heart, blood vessels, skin and eyes. It occurs most often between the ages of 50 and 59 [3]. Early diagnosis is key to the success of optimal treatment, especially for patients with significant risk factors such as high disease activity, the presence of autoantibodies, and early joint injury [4]. Although the outlook is now promising for most patients, many patients still do not respond to current treatments. Therefore, it is urgent to further elucidate the pathogenesis of RA in order to propose new treatment methods.

Pathogenetic changes in the synovial membrane are related to the persistent inflammation in RA [5]. On the one hand, macrophage-like synoviocytes (MLSs) secrete IL-1, IL-6, IL-26 and TNF-α [6]. On the other hand, fibroblast-like synoviocytes (FLSs) produce IL-6, IL-26, MMPs, prostaglandins and leukotrienes [7, 8]. In addition, infiltration of adaptive immune cells such as CD4^+^ memory T cells and B cells into the synovial sublining mediates damage and erosion formation in later disease [9, 10].

T cells also play an important role in RA. CD4^+^ T cells drive the occurrence and progression of RA by secreting IL-6 [11]. At the meantime, both IFN-γ-expression Th1 cells and IL-17-producing helper T (Th17) cells also have important roles in RA development [2]. Th17 cells and their effector molecules interleukin17, interferon (IFN) γ, tumor necrosis factor (TNF) α, and granulocyte-macrophage colony-stimulating factor (GM-SCF) are involved in the pathology of RA [12]. Several studies have shown that IL-17A is involved in various pathological processes of RA, such as activation of fibroblast-like synovial cells (FLS) [13], maturation and function of osteoclasts [14], recruitment and activation of neutrophils [15], macrophages [16], and B cells. Clinical trials of IL-17A blocking treatment for RA have been conducted. IL-17A blocking improved the signs and symptoms of RA despite the inadequate therapeutic response [17]. However, the role of CD8^+^ T cells in RA is still controversial. Some studies suggest that these cells may have a major pro-inflammatory role in the disease, others suggest the opposite [18, 19]. Therefore, it is very important to evaluate the infiltration of immune cells, search for new biomarkers to clarify the molecular mechanism of RA and find new therapeutic targets from the perspective of immune system.

In this work, we collected 25 HC and 111 RA samples for analyzing differentially expressed genes and further conducting GESA analysis. WGCNA was utilized to explore the gene modules and CD8^+^ T cell-related genes (CRGs) were chosen for PPI, KEGG and GO analysis. Besides, to explore biomarkers of RA in depth, machine learning algorithms and bioinformatics analysis were performed. Next, ssGSEA algorithms were adopted to investigate the differences in immune infiltration of 22 immune cell subsets between RA and healthy control (HC) group. Finally, optimal classification analysis based on consensus clustering combined with consensus clustering algorithms were conducted.

## MATERIALS AND METHODS

### Microarray download and differential expression analysis

We collected a total of four transcriptome datasets from the Gene Expression Omnibus (GEO) database that contained samples from both patients with rheumatoid arthritis (RA) and healthy control (HC), including GSE1919 (platform: GPL91), GSE48780 (platform: GPL570), GSE55235 (platform: GPL96), and GSE55457 (platform: GPL96). Following the exclusion of non-standard samples, we obtained a final sample size of 25 HC and 111 RA samples for analysis. To ensure accurate gene annotation, we employed Perl scripts to annotate gene names based on the platform file of each GEO dataset. Furthermore, to mitigate any potential batch effects, we utilized the “SVA” script in the R environment to standardize the data across the four independent GEO datasets [20, 21]. Subsequently, we used the “limma” script to perform differential analysis on the transcriptional matrix of the HC and RA groups. The criteria for selecting differentially expressed genes were set at |FC| ≥ 2 and an adjusted *p*-value less than 0.05. Finally, using the KEGG pathway reference gene set “c2.cp.kegg.v7.4.symbols.gmt”, we conducted Gene Set Enrichment Analysis (GSEA) based on the fold change in gene expression between the two groups.

### Screening for the gene modules most relevant to immune cells

We utilized the transcriptional matrix and immune cell scores of the samples to construct a Weighted Gene Co-expression Network Analysis (WGCNA) model in order to identify the gene modules most strongly correlated with immune cells, using the “WGCNA” script. The immune cell scores of both HC and RA samples based on 23 marker genes for immune cells were evaluated using the single-sample Gene Set Enrichment Analysis (ssGSEA) algorithm. Based on the transcriptional data of each sample, we subsequently constructed a clustering tree model to determine and exclude outlier samples. We further constructed a free-scale network based on the optimal soft threshold value (β) and employed the dynamic tree to cut and integrate the obtained gene modules into similar ones. We utilized the Pearson correlation algorithm to calculate the correlation of transcriptional data in each gene module. Finally, based on the Pearson correlation calculation of the immune cell scores of 23 types of immune cells and the correlation between genes in each independent gene module, we selected the gene module that was most relevant for subsequent analysis.

### Identification of differential expression CD8^+^ T cell related genes (CRGs) and potential function analysis

To identify the differentially expressed CRGs (DE-CRGs), we employed a Venn diagram to determine the intersection of the gene sets obtained from differential expression analysis and WGCNA analysis. Using the STRING database, we predicted the protein-protein interactions (PPI) among the DE-CRGs. Moreover, we utilized the “clusterProfiler” script to predict the Gene Ontology (GO) and Kyoto Encyclopedia of Genes and Genomes (KEGG) pathways that were associated with the DE-CRGs.

### Development of machine learning to screen for CRGs biomarkers

In our study, we employed two machine learning algorithms to identify potential biomarkers that were associated with RA. Firstly, we utilized the “randomForest” script to calculate the importance score for each DE-CRG, and we considered variables with a threshold greater than 1 to be important. Additionally, we built a LASSO model using the “glmnet” script to identify key variables based on the optimal coefficient and minimum lambda value. The intersection of genes that were obtained from the two different machine learning algorithms was considered as potential biomarkers for RA.

### Diagnostic effectiveness evaluation of CRGs biomarkers

Using the “ggplot2” and “limma” scripts, we performed an analysis of the expression of CRG biomarkers in both the HC and RA groups. Subsequently, we utilized the “pROC” script to draw ROC curves and evaluate the AUC values for each CRG biomarker. In addition, we constructed a nomogram diagnostic model based on the expression profiles of the CRG biomarkers, using the “rms” script. For each sample, we calculated the nomogram score using the following formula: Nomogram score = GDF15 × −1.23 + IGHM × 0.57 + IGLC1 × 0.25.

### Molecular subtype and immune infiltration characteristic analysis

In this study, we conducted a consensus clustering analysis of RA samples using the “ConsensusClusterPlus” script to identify different molecular subtypes. Based on the optimal classification of k = 2–9, the RA samples were successfully classified into two distinct molecular subtypes. To further understand the immune infiltration characteristics of these subtypes, we employed the ssGSEA algorithm to calculate the quantitative data for immune infiltration based on the expression of 23 immune cell marker genes in the HC and RA groups, and used the ggplot2 script to visualize the results. Additionally, we used Pearson correlation analysis to investigate the potential relationship between CRG biomarkers and immune infiltration features, and only considered *p*-values less than 0.05 to be statistically significant.

### RT-qPCR and Western blot

Total RNA of clinical tissues was extracted and been reversed transcription into cDNA using EasyScript^®^ First-Strand cDNA Synthesis SuperMix (TransGen Biotech). Real-time qPCR in the BioRad CFX96 Touch system was conducted with SYBR Green (Supplementary Table 1). All results are relative to Actin expression. Each assessment was conducted in three biological replicates. Clinical tissues are lysed and the concentration of protein was measured. Equal amount of protein was dissolved in the gel and immunoblot was performed with the indicated primary antibodies using 8% or 10% polyacrylamide.

### Statistical analysis

In this study, the R and Perl languages environment were utilized for data extraction and preprocessing. The Pearson algorithm was employed to calculate the correlation between two groups, while the Wilcoxon rank-sum test was utilized to assess statistical differences between two groups, and ANOVA was utilized to evaluate statistical differences between multiple groups. A *p*-value of less than 0.05 was considered statistically significant. Data were expressed as mean ± standard deviation (SD), and statistical significance was indicated by ^*^p < 0.05, ^**^p < 0.01, and ^***^p < 0.001.

## RESULTS

### Differential expression and GSEA enrichment analysis

**Figure 1:**
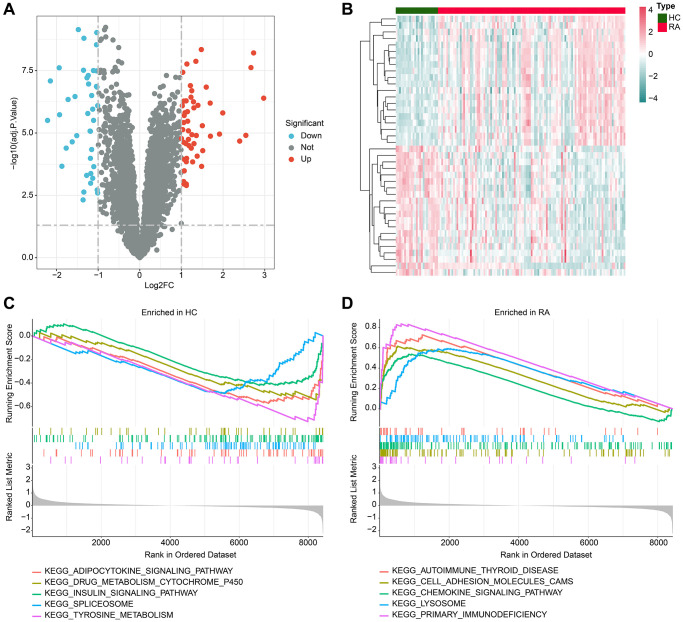
**Analysis of differential gene expression and GSEA.** (**A**) Volcano plot shows the differential gene expression between the HC and RA groups. The screening threshold is set at |FC|≥2 and adj.*p* value < 0.05, where red dots represent upregulated genes, and blue dots represent downregulated genes. (**B**) Heatmap of the differential gene expression in the HC and RA groups. (**C**, **D**) Enrichment analysis of GSEA pathway based on differential gene expression in HC and RA groups.

In this study, we performed a comprehensive evaluation by utilizing 25 HC and 111 RA samples that were obtained from four independent Gene Expression Omnibus (GEO) datasets, namely GSE1919, GSE48780, GSE55235, and GSE55457. A stringent filtering criterion was applied with a threshold set at |FC|≥2 and adj.*p* value <0.05, followed by utilizing the “limma” script to identify the differentially expressed genes (DEGs) between the HC and RA groups. Our differential expression analysis revealed a total of 92 DEGs, comprising 40 downregulated genes and 52 upregulated genes (Figure 1A, 1B). Furthermore, our gene set enrichment analysis (GSEA) results demonstrated significant enrichment of adipocytokine signaling pathway, spliceosome, and tyrosine metabolism pathways in the HC group. Interestingly, in the RA group, a significant enrichment of immune-related signaling pathways, such as cell adhesion molecules (CAMs), chemokine signaling pathway, lysosome, and primary immunodeficiency, was observed (Figure 1C, 1D).

### Identification of key immune-related gene modules for RA via WGCNA

**Figure 2:**
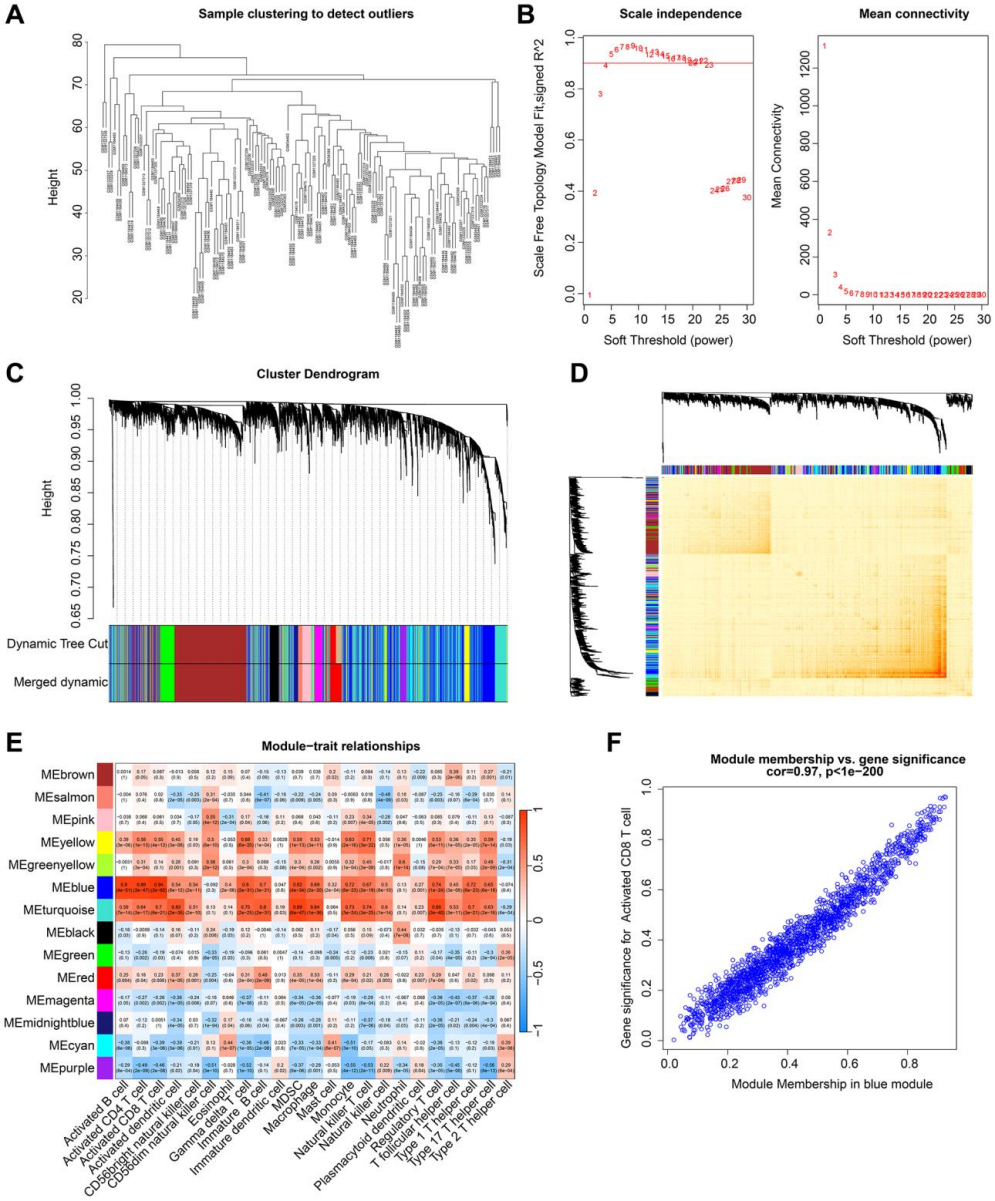
**Development of WGCNA to identify the gene modules most correlated with immune cells in RA.** (**A**) Cluster analysis of samples. (**B**) Construction of a scale free network with a soft threshold (β) set to 4. (**C**) Identification of gene modules based on dynamic tree cutting. (**D**) Correlation analysis of gene expression between different gene modules. (**E**) Correlation analysis of gene modules and 23 types of immune cells. Blue represents negative correlation and red represents positive correlation. (**F**) Association between module members and gene significance.

WGCNA was utilized in this study to further explore the gene modules that were primarily associated with the immune system in RA. To eliminate outliers, clustering analysis was performed on all samples using the expression matrix data (Figure 2A). A scale-free network was constructed by setting a soft threshold (β) of 4 (Figure 2B). The dynamic tree cutting method was used to categorize gene modules and integrate similar ones, resulting in a total of 14 gene modules being identified for further analysis (Figure 2C). The heatmap of gene modules indicated a potential correlation between each module (Figure 2D). To determine the correlation between gene modules and 23 types of immune cells, Pearson correlation algorithm was used. The outcomes showed that different gene modules were significantly correlated with 23 types of immune cells, with the blue module demonstrating the most significant correlation with CD8^+^ T cells (r = 0.94, *p* = 2e-62) (Figure 2E). A scatter plot revealed a strong and significant correlation (r = 0.97, *p* < 1e-200) between CD8^+^ T cell-related genes and the blue module (Figure 2F). Therefore, the genes in the blue module were recognized as CD8^+^ T cell-related genes (CRGs) and were included in subsequent analyses.

### Identification and functional enrichment analysis of differential CRGs

**Figure 3:**
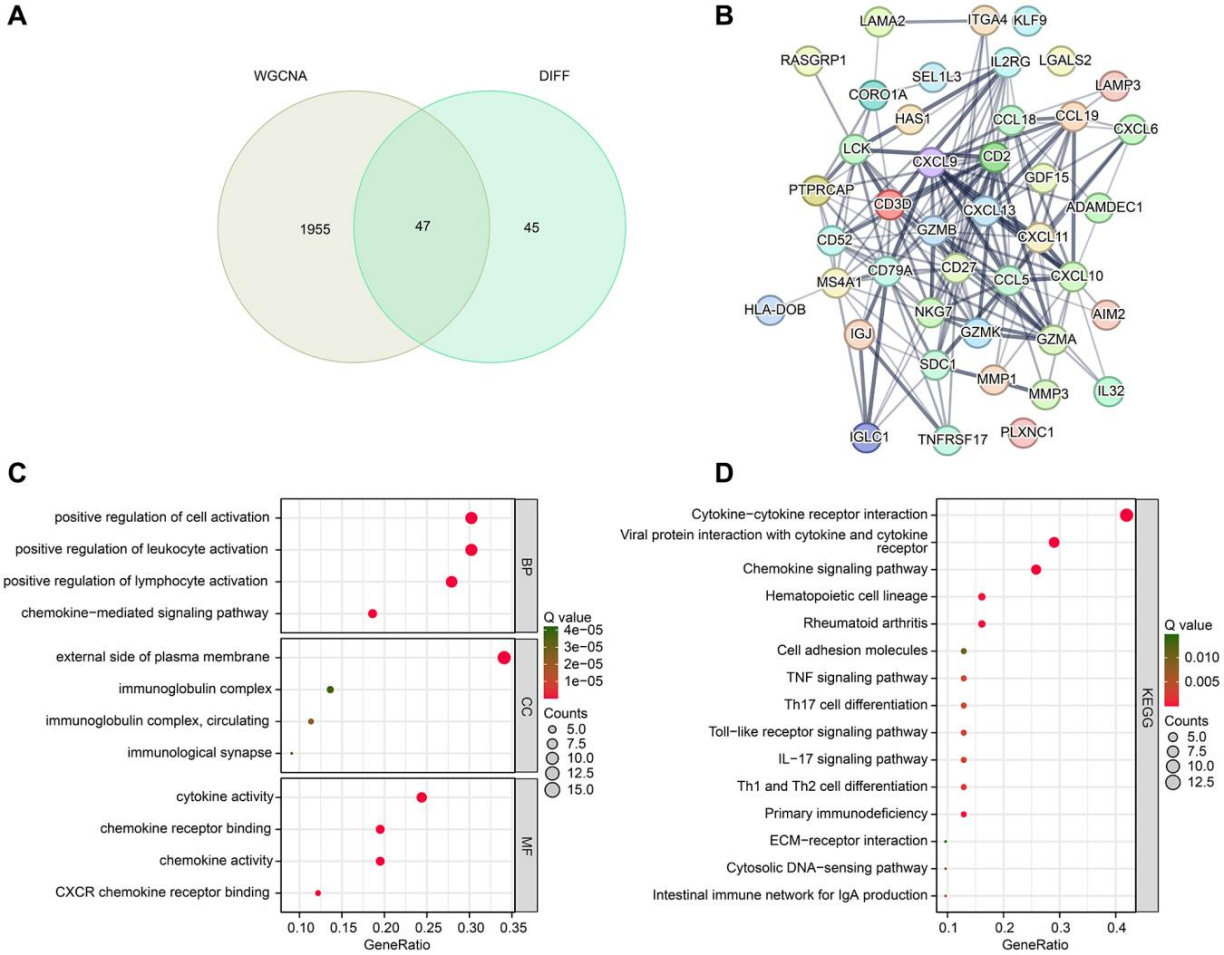
**Identification of DE-CRGs and potential molecular mechanism investigation.** (**A**) Identification of DE-CRGs based on WGCNA and differential expression analysis. (**B**) PPI network reveals the potential interaction between the DE-CRGs. (**C**, **D**) GO and KEGG enrichment analysis of 47 DE-CRGs.

The study integrated the results of WGCNA and differential expression analysis to identify 47 DE-CRGs (Figure 3A). Protein-protein interaction (PPI) analysis revealed significant correlations among these DE-CRGs (Figure 3B). To gain further insights into the potential molecular mechanisms of DE-CRGs in RA, we conducted GO and KEGG enrichment analyses. The GO analysis results suggested that the DE-CRGs were significantly enriched in immunological functions, such as positive regulation of cell activation, positive regulation of leukocyte activation, external side of plasma membrane, and cytokine activity (Figure 3C). The KEGG analysis results indicated that the CRGs were significantly enriched in immunological signaling pathways, such as cytokine-cytokine receptor interaction, viral protein interaction with cytokine and cytokine receptor, and chemokine signaling pathway (Figure 3D).

### Exploration of CRGs-related biomarkers for RA

**Figure 4:**
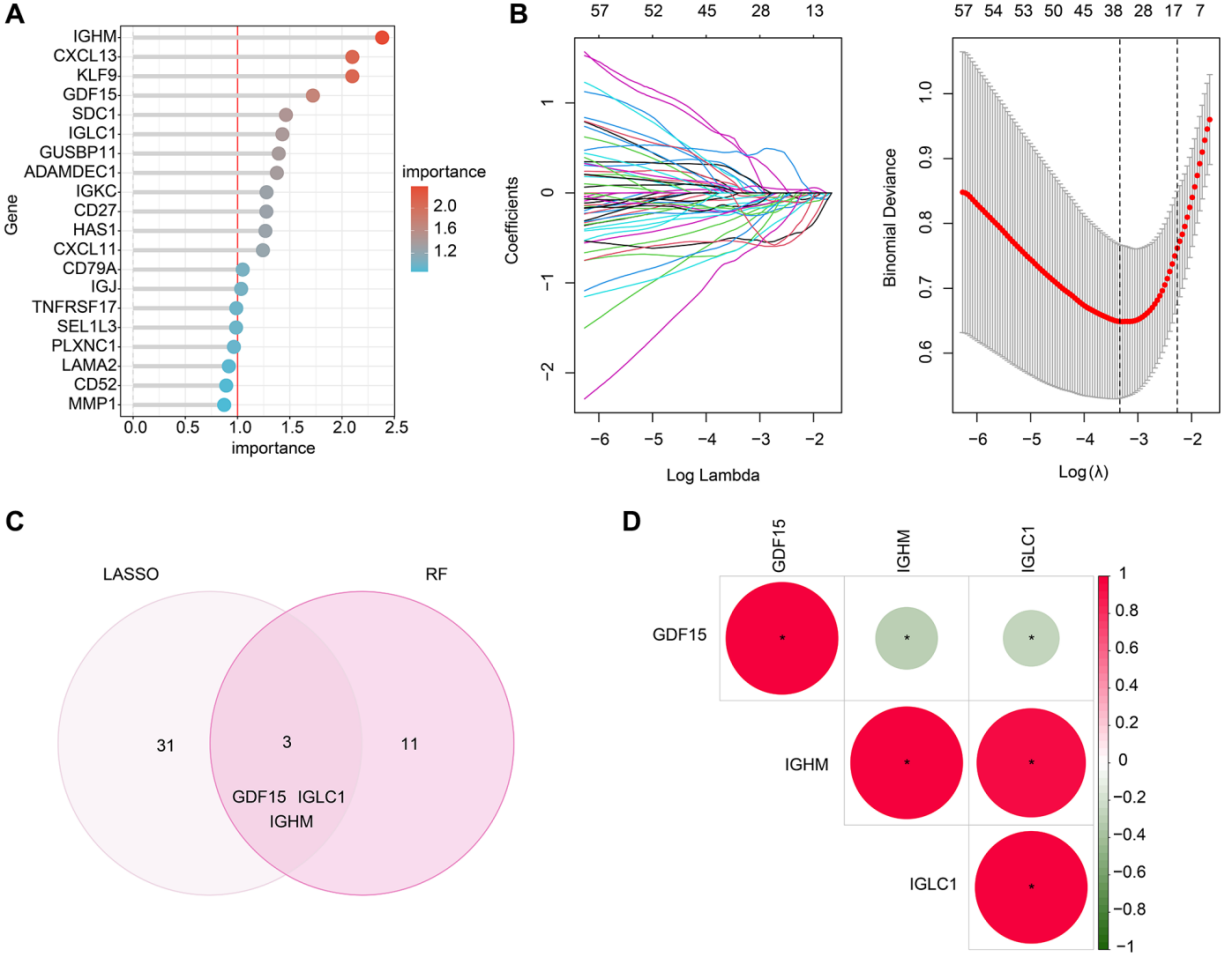
**Screening the CRGs-related biomarkers based on the machine learning algorithm.** (**A**) RF algorithm shows the importance of CRGs, which the cutoff of importance is set at 1. (**B**) The coefficients and log lambda value distribution of CRGs based on LASSO model. (**C**) Identification of CRGs- related biomarkers based on the LASSO and RF algorithms. (**D**) Correlation analysis between GDF15, IGHM and IGLC1.

In order to further investigate the potential biomarkers associated with CRGs in RA, we employed two machine learning algorithms. Specifically, we utilized the RF algorithm to identify a set of 14 crucial CRG variables (Figure 4A). Furthermore, the LASSO analysis to identify 34 significant feature variables was conducted based on the optimal coefficients and minimum lambda values of the model (Figure 4B). By combining the results of the RF and LASSO analyses, we successfully shortlisted three biomarkers linked to CRGs, namely GDF15, IGLC1 and IGHM (Figure 4C). Our correlation analysis subsequently showed a significant negative correlation between GDF15 and IGLC1 and IGHM, while IGLC1 was positively correlated with IGHM, as demonstrated in Figure 4D.

### Diagnostic effectiveness assessment of CRGs-related biomarkers

**Figure 5:**
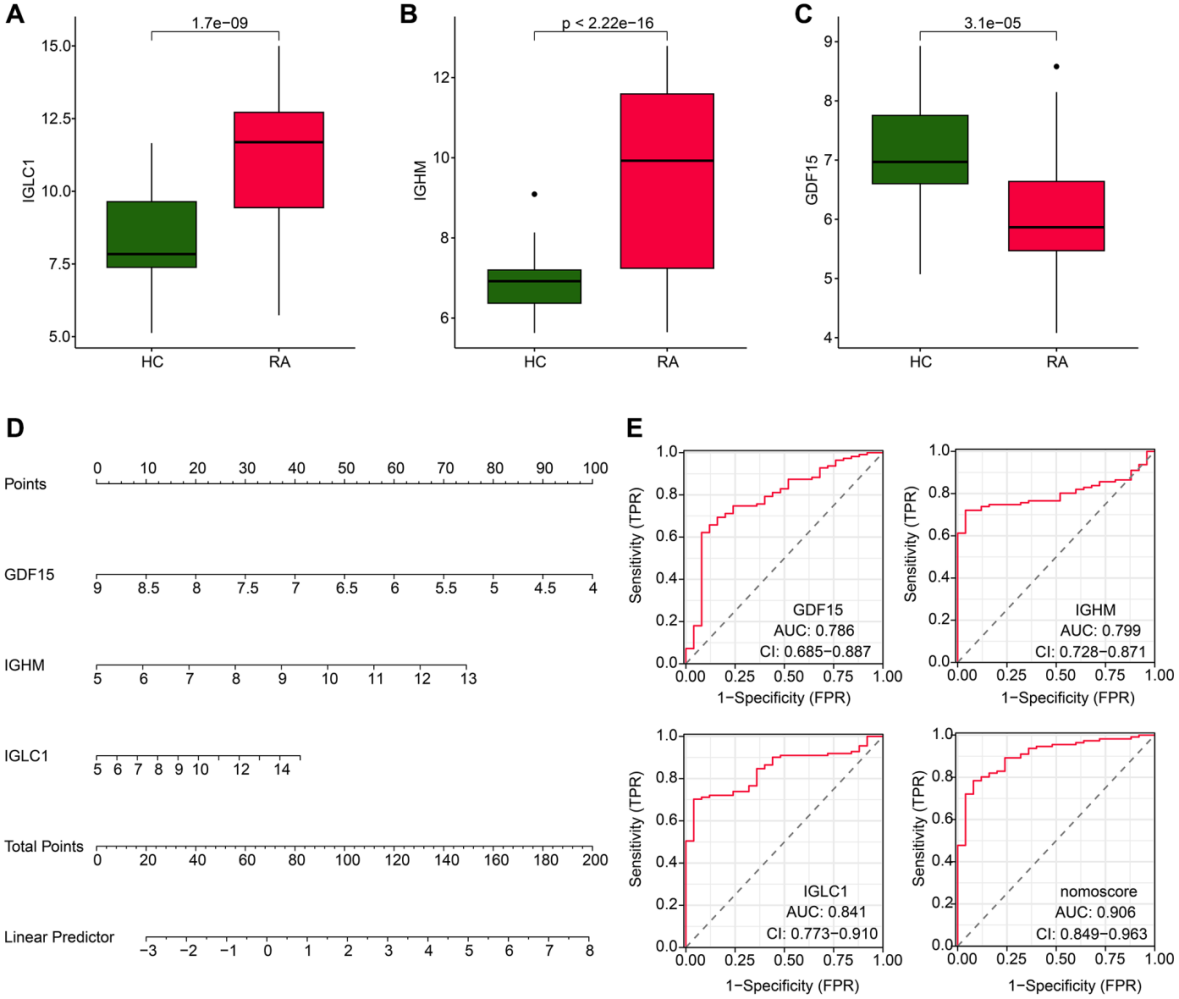
**Expression level and nomogram construction of CRGs for RA.** (**A**–**C**) The expression analysis of IGLC1, IGHM and GDF15 in HC and RA groups. (**D**) Nomogram development of IGLC1, IGHM and GDF15. (**E**) Diagnostic effectiveness exploration of IGLC1, IGHM, GDF15 and nomogram score.

Further investigations were conducted to explore the diagnostic potential of three biomarkers associated with CRGs for RA. The findings of our expression level analysis demonstrated that the RA group exhibited significantly higher expressions of IGLC1 and IGHM, whereas the expression of GDF15 was lower compared to the HC group (Figure 5A–5C). In addition, the expression profiles of these three biomarkers were utilized to develop a nomogram, which could effectively evaluate the diagnostic accuracy of RA (Figure 5D). The ROC curve analysis indicated that the AUC of IGLC1, IGHM, and GDF15 was 0.841, 0.799, and 0.786, respectively. It is noteworthy that the AUC of the nomogram was 0.906, which was significantly higher than that of the individual CRGs- related biomarkers (Figure 5E).

### Immune infiltration characteristic analysis

**Figure 6:**
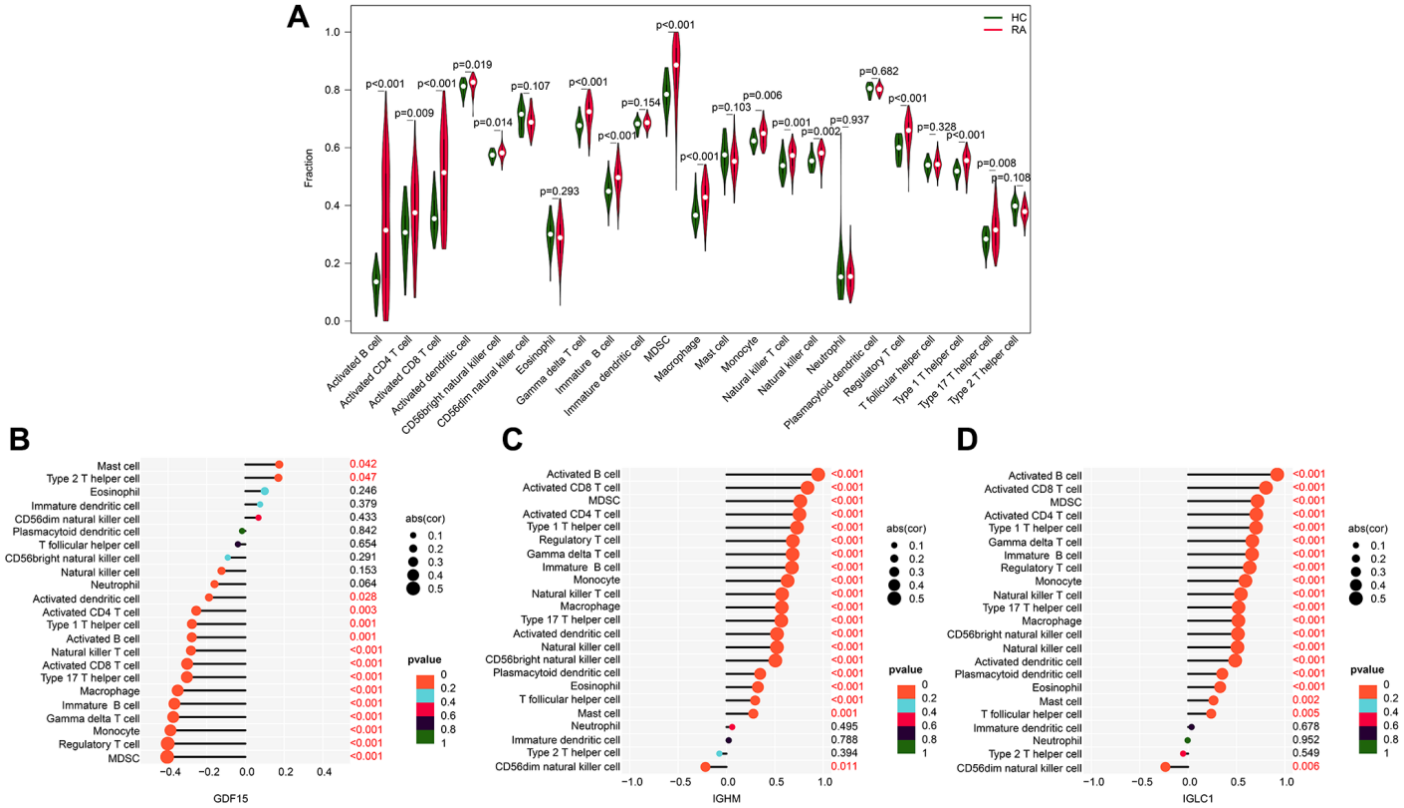
**Association exploration of CRGs-related biomarkers and immune infiltration characteristic.** (**A**) Relative quantities of 23 immune cells in the HC and RA groups. (**B**–**D**) Correlation analysis of IGLC1, IGHM, GDF15, and 23 immune infiltration features.

We conducted further investigations to explore the potential relationship between the biomarkers associated with CRGs and immune infiltration characteristics. We observed a higher immune infiltration status in the RA group compared to the HC group, with increased levels of activated B cells, CD4^+^ T cells, CD8^+^ T cells, MDSCs, and natural killer T cells (Figure 6A). Our correlation analysis of the CRGs-related biomarkers and immune infiltration characteristics revealed that GDF15 was positively correlated with mast cells and type 2 T helper cells, but negatively correlated with most other immune cells. On the other hand, IGHM and IGLC1 were negatively correlated with CD56dim natural killer cells, while positively associated with other immune cells (Figure 6B–6D). These findings illustrate the potential association between CRG-related biomarkers and immune infiltration features of RA, providing a novel perspective for exploring the underlying mechanisms of RA.

### Molecular subtype characterization based on CRGs-related biomarkers

**Figure 7:**
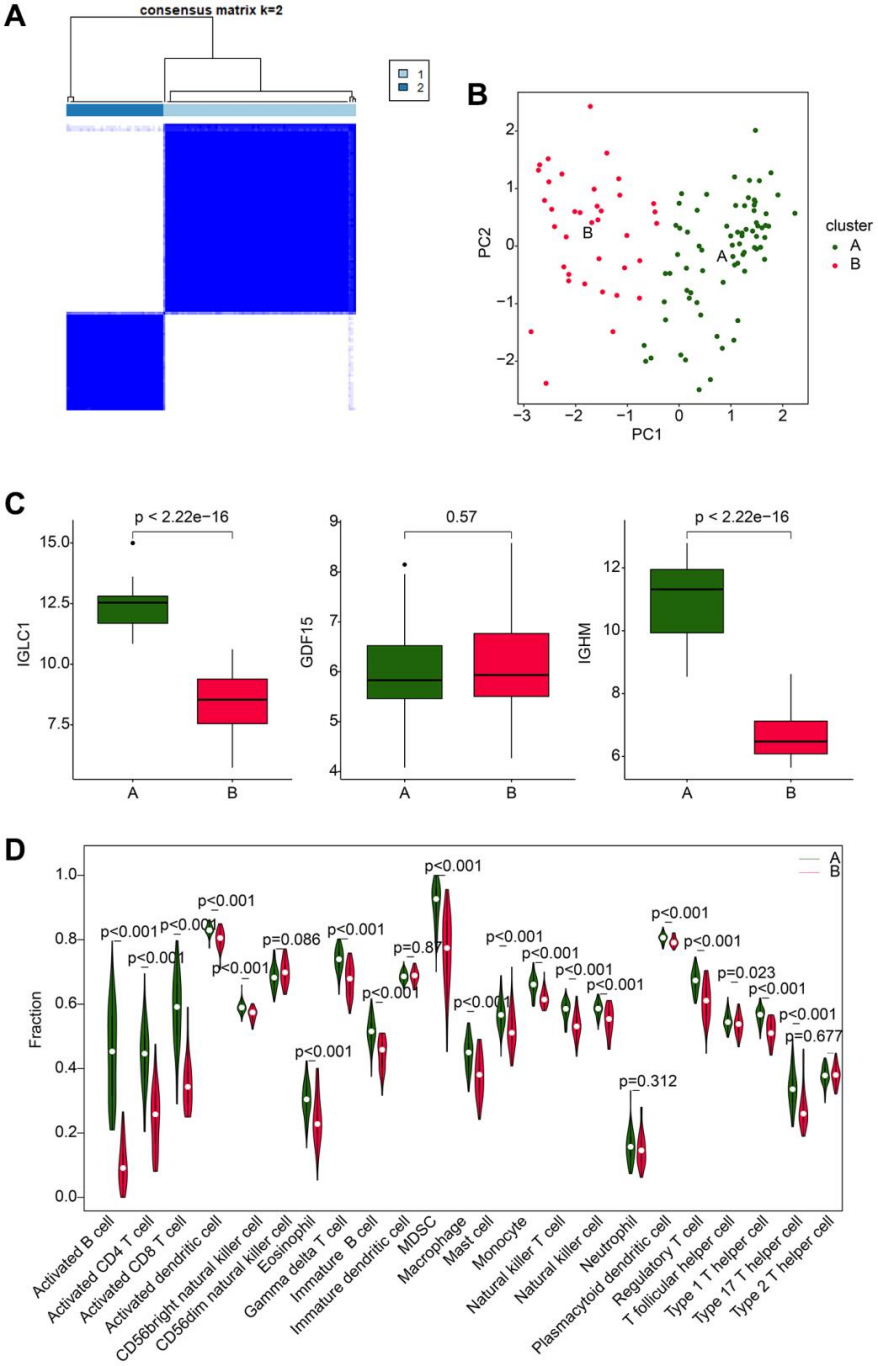
**Molecular subtype analysis based on CRGs.** (**A**) Optimal classification analysis based on consensus clustering of CRGs. (**B**) PCA analysis of different molecular subtypes of RA. (**C**) Expression analysis of IGLC1, IGHM, and GDF15 in different molecular subtypes. (**D**) Analysis of immune infiltration characteristics in molecular subtypes of RA samples.

Using the expression profiles of IGLC1, IGHM, and GDF15, we thoroughly classified RA samples into distinct molecular subtypes and analyzed the immune infiltration characteristics between the subtypes. The optimal clustering k = 2, based on consensus clustering analysis, was applied to categorize the RA samples into two distinct molecular subtypes, with subtype A comprising of 74 samples and subtype B comprising of 37 samples (Figure 7A). The unsupervised PCA results demonstrated that the molecular subtypes exhibited independent distribution patterns, which signified significant differences between the two analyzed subtypes of RA (Figure 7B). Remarkably, the expression profiles of IGLC1, IGHM, and GDF15 indicated that the expression of IGLC1 and IGHM was substantially reduced in subtype B compared to subtype A, while there was no significant difference in the expression of GDF15 between the two groups (Figure 7C). The quantitative results of immune infiltration characteristics revealed that RA samples in subtype A exhibited a higher immune state, with significantly increased content of immune cells such as activated B cell, CD4^+^ T cell, CD8^+^ T cell, and activated dendritic cell compared to subtype B (Figure 7D). These findings suggest that RA can be precisely stratified into distinct molecular subtypes based on CRGs-related biomarkers, which are likely to be associated with immune infiltration.

### IGLC1, IGHM, and GDF15 expression level in RA samples

**Figure 8:**
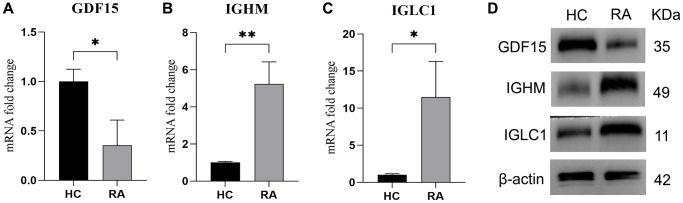
**Expression level of IGLC1, IGHM, and GDF15.** (**A**–**C**) RT-qPCR was used to test the mRNA level of GDF15 (**A**), IGHM (**B**) and IGLC1 (**C**) in HC and RA samples. (**D**) The protein level of IGLC1, IGHM, and GDF15 in HC and RA samples.

15 HC samples and 15 RA samples were collected to confirm the expressions of selected biomarkers in RA patients. The mRNA level of GDF15 was lower in RA samples (Figure 8A), while the mRNA level of IGHM (Figure 8B) and IGLC1 (Figure 8C) were higher in RA samples compared with HC samples. The same results were also observed in protein level (Figure 8D).

## DISCUSSION

As an autoimmune disease, the dysfunction of immune cells plays an important role in the occurrence of RA. Despite significant advances in current therapies, there are still a lot of patients who do not respond to current therapies. Therefore, elucidating the pathogenesis of RA is important. Here, we attempt to investigate the biomarkers and draw the infiltration patterns of RA, which can not only provide reference for clinical diagnosis, but also provide new therapeutic targets for drug development.

In our study, we identified a total of 92 DEGs, including 40 risen and 52 declined DEGs. Then GSEA analysis revealed significant enrichment of adipocytokine signaling pathway, spliceosome, and tyrosine metabolism pathways in the HC group. But in the RA group, a significant enrichment of immune-related signaling pathways, such as cell adhesion molecules (CAMs), chemokine signaling pathway, lysosome, and primary immunodeficiency was observed. This suggested that the immune system was activated in RA, accompanied by a large number of immune cell infiltration. This is consistent with the conclusion that RA, an autoimmune disease, is closely related to imbalance of the immune system [22, 23].

Furthermore, we investigated the gene modules that were primarily associated with the immune system in RA. We identified a total of 14 gene modules, and determined the correlation between the gene modules and 22 kinds of immune cells. The results showed that the blue module had the most significant correlation with CD8^+^ T cells. As previously reported, the absolute number of CD8^+^ T cells in the peripheral blood of patients with early RA was higher than that of healthy controls [24], which indicated the important role of CD8^+^ T cells in RA. Next, we picked 47 DE-CRGs for PPI, KEGG and GO analysis and the results show robust immune activation in RA.

Moreover, two machine learning algorithms were employed to analyzed the biomarkers. The RF algorithm to identify a set of 14 crucial CRG variables and the LASSO analysis to identify 34 significant feature variables. By combining the results of the RF and LASSO analyses, GDF15, IGLC1, and IGHM were picked out. At the meantime, high levels of GDF15, IGLC1, and IGHM were observed in RA. These results suggest that the three genes may contribute to the development of RA. Previous research has shown that GDF15 serum levels might be a biomarker to predict high RA disease activity [25], which could partly prove the accuracy of our analytical model. However, their molecular function in RA needs to be further verified.

CD8^+^ T cells is up-regulated proportionally in RA and have the characteristics of secreting inflammatory mediators [26]. Some reports suggest that CD8^+^ T cells promote the progression of RA by releasing pro-inflammatory and cytolytic mediators [26, 27]. However, due to the complexity of RA disease, there are still conflicting results in experimental studies of CD8^+^ T cells in RA. Coupled with the scarcity of data, the function of CD8^+^ T cells in the RA immune-infiltrating microenvironment remains to be further explored [18]. In addition to Th1, Th17 and macrophage, previous studies have shown that IL-10 secreted by Th2 promotes antibody production in RA [28] and NK cells might play an important role in bone destruction in RA [29]. To explore the potential relationship between CRGs-related biomarkers and immune infiltration characteristics, correlation analysis was performed between CRGs-related biomarkers and immune infiltration cells. The results showed that GDF15 was positively correlated with mast cells and T helper cells type 2, but negatively correlated with most other immune cells. On the other hand, IGHM and IGLC1 were negatively correlated with CD56dim natural killer cells, but positively correlated with other immune cells. These results suggest that these three CRGs-related biomarkers may be particularly important for immune system imbalances.

Finally, optimal classification analysis was conducted to classified RA samples into distinct molecular subtypes. Compared with subtype A, the expression of IGLC1 and IGHM in subtype B was significantly decreased, while there was no significant difference in GDF15. Immune infiltration characteristics analysis showed that the immune status of RA samples of subtype A was higher, and the contents of activated B cells, CD4^+^ T cells, CD8^+^ T cells, activated dendritic cells and other immune cells were significantly increased compared with those of subtype B. These findings clue that RA can be accurately stratified into different molecular subtypes based on CRGs-related biomarkers, which indicate the reliability of the three genes as RA biomarkers and provide guidance for personalized therapy.

Although we screened out three targets through machine learning methods and verified their abnormal expression levels through clinical specimens. However, due to limited conditions, we were not able to conduct further studies on the biological functions of the selected targets. Additionally, the correlation analysis results obtained by machine learning need to be further studied on causality. Further analyze of these three targets in future studies will be of great significance on RA.

## CONCLUSION

In brief, this study reported that abnormal activation and imbalance of immune cells in RA, and identified GDF15, IGLC1, and IGHM as diagnostic markers of RA. In addition, GDF15 was positively correlated with mast cells and type 2 T helper cells, but negatively correlated with most other immune cells. On the other hand, IGHM and IGLC1 were negatively correlated with CD56dim natural killer cells, while positively associated with other immune cells. Finally, RA samples in subtype A exhibited a higher immune state, with significantly increased content of immune cells. This study can provide guidance for the discovery of drug targets and personalized therapy for RA patients.

## Supplementary Materials

Supplementary Table 1
