# Supplementary material for: Identification of CD8+ T cell-related biomarkers and immune infiltration characteristic of rheumatoid arthritis
Source: Aging (Albany NY). 2024 Jan 16;16(2):1399–413. doi: 10.18632/aging.205435 (PMC10866417; doi:10.18632/aging.205435)
Supplement: Supplementary Table 1 [file aging-16-205435-s001.pdf]

SUPPLEMENTARY TABLE

Supplementary Table 1. The primer sequence for genes.

GDF15	Forward primer	GCAAGAACTCAGGACGGTGA
	Reverse primer	TGGAGTCTTCGGAGTGCAAC
IGHM	Forward primer	AGATGGTCTGCTTCAGTGGC
	Reverse primer	AGCTGTGAAAACCCACACCA
IGLC1	Forward primer	CCCACTGTCACTCTGTTCCC
	Reverse primer	CCGCGTACTTGTTGTTGCTC
